# Cholesterol Serum Levels and Use of Statins in Graves' Orbitopathy: A New Starting Point for the Therapy

**DOI:** 10.3389/fendo.2019.00933

**Published:** 2020-01-22

**Authors:** Giulia Lanzolla, Guia Vannucchi, Ilaria Ionni, Irene Campi, Federica Sileo, Elisa Lazzaroni, Michele Marinò

**Affiliations:** ^1^Endocrinology Units, Department of Clinical and Experimental Medicine, University of Pisa and University Hospital of Pisa, Pisa, Italy; ^2^Department of Endocrine and Metabolic Diseases, Istituto Auxologico Italiano Istituto di Ricovero e Cura a Carattere Scientifico, Milan, Italy; ^3^Department of Pathophysiology and Transplantation, University of Milan, Milan, Italy; ^4^Endocrinology and Metabolism Unit, Fondazione IRCCS Cà Granda, University of Milan, Milan, Italy

**Keywords:** Graves' orbitopathy, Graves' disease, thyroid, autoimmunity, statin, 3-hydroxy-3-methylglutaryl-coenzyme reductase, cholesterol, pleiotropic effects of statins

## Abstract

Graves' Orbitopathy (GO) is the most frequent extrathyroidal manifestation of Graves' disease (GD). Its ultimate cause remains unclear, but it is commonly considered an autoimmune disorder due to self recognition of autoantigens constitutively expressed by orbital fibroblasts (OFs), and thyroid epithelial cells. High dose intravenous glucocorticoids (ivGC) are the most commonly used treatment for moderately severe and active GO. However, based on the complex pathogenesis of GO, a number of factors may have a protective and maybe a therapeutic role. The use of other medications improving the effect of GC may increase the overall effectiveness of the therapy and reduce GC doses, thereby limiting side effects. Recently, a possible protective role of 3-hydroxy-3-methylglutaryl-coenzyme reductase inhibitors, the so-called statins, and perhaps of lowering cholesterol levels, has been proposed. Thus, statins have been reported to be associated with a reduced frequency of GO in GD patients and in recent cross-sectional and retrospective studies a significant correlation was found between the occurrence of GO and both total and LDL-cholesterol in patients with a GD of relatively recent onset, suggesting a role of cholesterol in the development of GO. Moreover, a correlation was found between the GO clinical activity score and total as well as LDL-cholesterol in untreated GO patients, depending on GO duration, indicating a role of cholesterol on GO activity. Therefore, statin treatment may be beneficial for GO. Here we review this subject, which offers new therapeutic perspectives for patients with GO.

## Introduction

Graves' orbitopathy (GO) is a disfiguring syndrome observed in patients with autoimmune thyroid diseases, especially Graves' disease (GD). Recognized risk factors for GO development are uncontrolled hyperthyroidism, radioiodine (RAI) treatment, and smoking. The pathogenesis of GO is due to a number of complex mechanisms which interplay among each other, namely humoral and cell-mediated immunity, cytokine production, and oxidative stress. Based on this knowledge, a number of factors may have a protective and maybe therapeutic role. Recently, a possible protective role of 3-hydroxy-3-methylglutaryl-coenzyme reductase inhibitors, commonly known as statins, and perhaps of lowering cholesterol levels, has been proposed. Here we review this subject which offers new therapeutic perspectives for patients with GO.

## Brief Notes on Go

GO is generally described as an autoimmune disease and it is observed in ~25–30% of patients with Graves' hyperthyroidism and less commonly in patients with hypothyroid autoimmune thyroiditis, or in those without thyroid dysfunction, the so-called euthyroid GO (1–3). Although the ultimate cause of GO remains unclear, some of the molecular mechanisms have been clarified. GO is likely due to an autoimmune reaction against autoantigens constitutively expressed by orbital fibroblasts (OFs) and thyroid epithelial cells, being the TSH-receptor the major autoantigen (1–3). Recently, a major role of the insulin-like growth factor-1 receptor (IGF-1R) has emerged (4). The extent to which IGF-1R has an intrinsic role or its actions are related to its capability of interplaying with the TSH-R has to be clarified (5). Overall, once the autoimmune reaction is started, both B and T cells infiltrate the fibroadipose orbital tissue and, through a number of interconnected events, lead to the cell modifications underlying the connective tissue remodeling typical of GO, with orbital fat expansion and extraocular muscle enlargement, which are ultimately responsible for the clinical manifestations of the disease (1, 2, 6).

The typical clinical features of GO are proptosis, inflammation, and diplopia. Subclinical eye involvement is common, whereas only ~3–5% of patients with GO have a severe disease characterized by corneal ulceration, severe inflammation, or compressive optic neuropathy (2, 7). In addition to genetic and demographical variables (6), the recognized risk factors associated with the development of GO in GD patients are age, sex, hypothyroidism, inadequate control of hyperthyroidism, radioiodine, and cigarette smoking (8–21).

The choice of treatment depends on the extent of GO activity, which is defined by a clinical activity score (1, 22). Patients affected with mild GO can be treated with lubricants and/or selenium (1, 23, 24), whereas, according to the European Group On Graves' Orbitopathy (EUGOGO) guidelines, high dose intravenous glucocorticoids GC (ivGC) are the first line treatment for moderately severe and active GO (1). However, more recently, new medications have been proposed, including rituximab (25), teprotumumab (26, 27), mycophenolate (28), and tocilizumab (29). Finally, orbital decompression, squint or palpebral surgery can be considered in patients with an inactive disease, for therapeutic or rehabilitative purposes (30). The use of systemic GC takes advantage from their immunosuppressive and anti-inflammatory actions, resulting in an overall beneficial effect ranging from ~35 to ~80% of patients (1, 31). Regarding the molecular mechanisms of GC, their actions on genomic and non-genomic pathways may affect the production of cytokines, the distribution of circulating T-cell subsets, the recruitment of monocytes and macrophages, the expression T-lymphocyte adhesion molecules, as well as the production, differentiation and homing of dendritic cells (32–34), thereby blocking the interplay between innate and adaptive immune responses. Unfortunately, patients are often selected to treatment because of the severity of the disease, despite having an inactive GO when immunosuppression is not effective. Thus, when patients with a long standing, still severe, but fibrotic and inactive GO are included in studies on immunosuppressive treatments, the response rate decreases remarkably. These observations may explain why about ~30% of patients do not respond to GC. Another potential explanation for the lack of response to treatment may be the necessity for a stronger immunosuppression. The use of additional medications may in theory improve the effects of GC, possibly increasing the overall effectiveness of the therapy and reducing GC dosage, thereby limiting side effects.

## Statins and Go

### Clinical Standpoint

Statins are the most commonly used drugs that improve cardiovascular risk by reducing low-density lipoprotein (LDL) cholesterol levels (26, 35). The JUPITER trial assessed the potential benefit of statins in healthy participants (36, 37), which was demonstrated by a significant reduction of markers of systemic inflammation (e.g., C-reactive protein), cholesterol levels, and the number of cardiovascular events (37, 38). The study showed the pleiotropic anti-inflammatory actions of statins, which were not related to their actions in terms of lowering cholesterol, and suggested that statins can be used as a new adjuvant anti-inflammatory therapy, in accordance to various studies (39–50). Statin treatment seems to be linked with the reduced risk of developing GO among patients with GD (51). Stein et al. studied a large cohort of patients with newly-diagnosed GD to identify risk factors associated with the development of GO, indicating that thyroidectomy as the chosen treatment of hyperthyroidism and exposure to statins may lower the risk (51). They investigated whether the administration of drugs with anti-inflammatory actions reduce the risk of GO. In a multivariate regression model, medical therapy with antithyroid medications, regardless of whether it was used alone or followed by RAI, did not modify the risk of GO compared to patients receiving RAI alone. In contrast, thyroidectomy alone or thyroidectomy with antithyroid medications were associated with a significant reduced risk of GO compared to RAI alone. Regarding TSH levels, individuals with TSH >7 μIU/mL had a higher risk of developing GO compared to those with TSH levels ≤7 μIU/mL, but this finding was not statistically significant. Finally, the authors assessed the potential protective effect on GO development of COX-2 inhibitors and statins. The use of statins for at least 60 days during an observation lasting 1 year was followed by a reduction of GO risk by ~40%. In contrast, statistically significant differences in terms of GO risk in patients exposed to non-statin cholesterol lowering medications were not found. Furthermore, in both univariate and multivariate models, no significant differences between subjects treated with COX-2 inhibitors and subjects not receiving these medications were observed (51). The protective action of statins on the risk of GO was considered as a direct effect of the immunomodulatory effects of statins (52).

### Molecular Mechanisms

**Figure 1:**
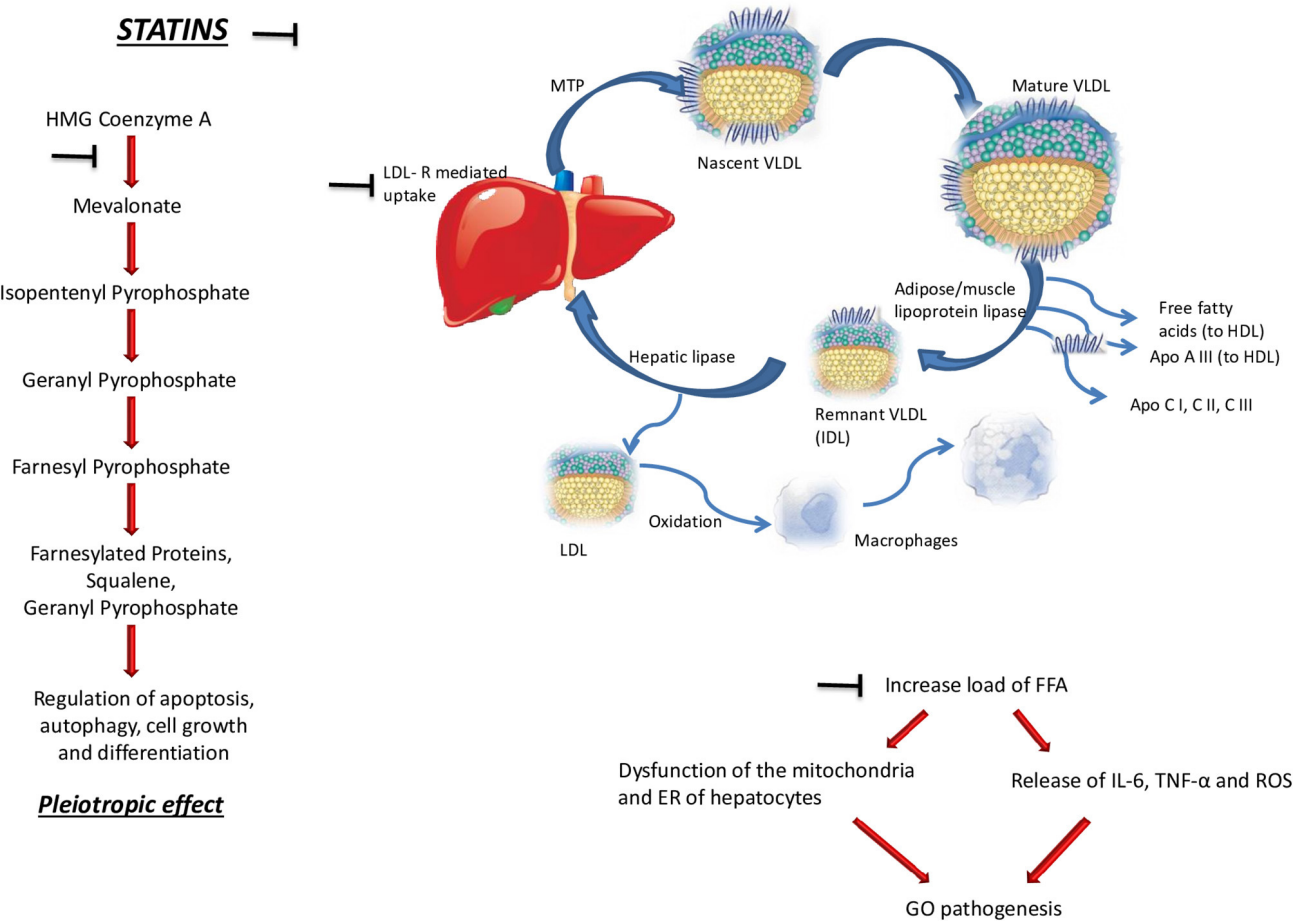
Molecular basis for a role of cholesterol and statins in the pathogenesis of Graves' Orbitopathy (GO). The symbol ⊣ indicates the pathways inhibited by statins, namely its pleiotropic effect in the regulation of apoptosis, autophagy, cell growth, and differentiation (left side of the figure), and the reduced synthesis of free fatty acids (upper sides of the figure). HMG, 3-hydroxy-3-methyl-glutaryl; MTP, microsomal triglyceride transfer protein; VLDL, very low density lipoproteins; HDL, high density lipoproteins; APO, apolipoprotein; LDL-R, low density lipoprotein receptor; FFAl, free fatty acids; TNFα, tumor necrosis factor α; ROS, reactive oxygen species; ER, endoplasmic reticulum.

As depicted in Figure 1, several studies reported that statins influence positively tissue remodeling and protect against heart failure (53, 54). The effects of statins are due to the competitive inhibition of 3-hydroxy-3-methyl-glutaryl-CoA (HMG-CoA) reductase, the key-limiting enzyme of cholesterol synthesis in the multistep mevalonate pathway in the liver (36, 37, 55). In addition to the biosynthesis of cholesterol, mevalonate is also a precursor of isoprenoids, namely farnesyl pyrophosphate (FPP) and geranylgeranyl pyrophosphate (GGPP), and the inhibition of the synthesis of isoprenoid intermediates plays a major role in most of the pleiotropic effects of statins (56). The prenyl groups derived by mevalonate are essential for cell activities, such as growth and differentiation, because of their role in the post-translational modification of proteins involved in intracellular signaling (57–59). Thus, it is demonstrated that statins induce cell death in many cell types, including macrophages (60, 61) cardiac myocytes (62), and smooth muscle cells (SMCs) (63–65). Moreover, the apoptosis induced by statins can be totally blocked by the addition of GGPP and partially by FPP, suggesting that geranylgeranylation is a key process for this effect which does not seem to be related with the reduction of cholesterol (66).

To investigate the effects of blocking the mevalonate pathway on both extrinsic and intrinsic apoptosis pathways, Ghavami et al. measured cysteine-dependent aspartate-directed proteases (caspase) cleavage in human atrial fibroblast (hATF) following simvastatin treatment for up to 120 h (67). Their data showed that the activation of the intrinsic pathway is selectively due to mevalonate cascade inhibition. In particular, through HMG-CoA inhibition, statins concomitantly induced autophagy, apoptosis, and unfolded protein response (UPR) stress in the endoplasmic reticulum (ER) in hATF (67). The initiation of apoptosis is driven by the disruption of the balance between pro- and anti-apoptotic proteins. HMG-CoA inhibition also promotes UPR/ER stress, as indicated by an increase in the ER of the chaperone and signaling regulator BIP/GRP78, and by the activation of endoplasmic reticulum kinase (PERK) and transcription factor 4 (ATF4), which in turn activate the CCAAT-enhancer-binding protein homologous protein CHOP to support apoptosis. Inhibition of HMG-CoA also leads to leak of cathepsin B (CATH-B) and L (CATH-L) from lysosomes, thereby enhancing apoptosis (67), and inducing the autophagy flux. These findings suggest that statins influence autophagic events acting on the co-regulation of apoptosis and UPR (67). Even if the molecular mechanisms are not fully clarified, the activity of statins on these processes may explain, at least in part, their pleiotropic effects also in GO. Thus, the autophagic and cell death programs, which influence the innate immune signaling pathway, are the key elements of the inflammation balance. The “danger hypothesis” and the intrinsic role of autophagy in GD have been recently described by Kawashima et al. (68), who suggested that autophagic events, through the processing and delivery of cytosolic antigens, enable thyroid cells to present antigens to CD4+ T cells. Furthermore, autophagy may influence the differentiation of OFs into mature adipocytes (69), and the early autophagic flux can also trigger the cell death-cascade in several cell types, including fibroblasts and monocytoid cells (70). Based on these assumptions, the association between statin treatment and an altered autophagy due to a severe reduction of protein prenylation, reflecting blocking of the mevalonate pathway, may be a potential explanation for their protective effect on GO. As reported above, statins induce cell death in human fibroblasts (67), and it would be interesting to investigate whether this effect can also influence the production of glycosaminoglycan by OFs, and their differentiation into adipocytes and myofibroblasts. Recently, Shih et al. reported a direct correlation between mast cells and macrophage infiltration in the Muller's muscle and the extent of the upper lid retraction (71). By Masson's trichrome staining, they evaluated the proportions of fat, muscle, and fibrosis in Muller's muscle specimens. Normal muscle samples had undulating bundles of smooth muscle fibers, a thin layer of connective tissue and small deposits of fat. In contrast, GO Muller's muscles presented a decreased muscle volume and an increase in fat tissue and fibrosis (71). The immunohistochemical study of Muller's muscle showed that normal samples were characterized by mast cells and macrophages infiltrating muscular tissue. In patients with GO the mast cell component was decreased, and the share of macrophages infiltrating Muller's muscle was significantly higher. To investigate to what extent the decrease in muscle reflected an increase in apoptosis, Shih et al. performed an immunohistochemical staining for caspase-3, which was undetectable in all samples, indicating that apoptosis is not increased and that the reduced muscle volume in GO Muller's muscles likely reflects an attenuated myogenesis, augmented adipogenesis, and transdifferentiation of myoblasts into adipocytes (71). The regression models showed that mast cell infiltrating Muller's muscle correlated positively with fractional fat composition, and negatively with fractional fibrosis. Moreover, a direct correlation between macrophage counts and both fibrosis and extent of the upper eyelid retraction was observed. Overall, the findings led to the conclusion that the degree of inflammatory cell infiltration of Muller's muscle is associated with the clinical severity of the upper eyelid retraction (71). Croons et al. demonstrated that statins have the capability of triggering apoptosis in macrophages, at concentrations at which SMCs are in contrast resistant (66). Thus, according to the ability of statins of affecting macrophage viability *in vitro* by inducing apoptosis, the early autophagic flux induced by statins may explain apoptosis of macrophages infiltrating Muller's muscles in GO patients, suggesting a beneficial effect of these medications.

A recent study by Shahida et al. showed that Simvastatin may inhibit adipogenesis in preadipocytes and human OFs, modulating the expression of early, and late adipogenic genes in both cell types (72). They used 3T3-L1 preadipocytes and human OFs, exposing them to 10% cigarette smoke extract (CSE) with or without Simvastatin, and compared gene expression between these cells and unexposed cells. In 3T3-L1 preadipocytes, Cyr61, Ptgs2, Erg1, and Zfp36 levels were greater in cells exposed to CSE compared with unexposed cells. Interestingly, Simvastatin downregulated the expression of these genes. Moreover, CSE alone did not induce adipogenesis, while Scid1, PPAR-γ, and adipogenesis itself were reduced in preadipocytes treated with Simvastatin compared to untreated cells. Similar effects were seen also in human OFs (72).

In addition to the above-mentioned actions, statins seem to have, at least *in vitro* and in experimental animal models of autoimmune diseases, an immunoregulatory action (73–76), whereas, to our knowledge, there are no reports on the use of statins in human autoimmune diseases. The main immunoregulatory action of statins seems to be related to tolerogenic dendritic cells (TolDCs), a specialized subset that induces immune tolerance and counteracts autoimmune responses (74). Atorvastatin, has been shown to induce TolDCs, thereby ameliorating experimental autoimmune diseases such as myasthenia gravis and experimental autoimmune encephalomyelitis (74, 75). In addition, statins may exert an inhibitory action on antigen-presenting dendritic cells. Thus, Simvastatin was found to inhibit the maturation of myeloid dendric cells derived from peripheral blood mononuclear cells from patients with autoimmune optic neuritis and to counteract the proliferation of T-cells induced by dendritic cells (76).

## Cholesterol and Go

In addition to a possible direct action of statins on the eye by their pleiotropic effects, the effects of these drugs in GO may also reflect lowering of cholesterol serum level (77). In a recent cross-sectional study, a significant correlation was found between the occurrence of GO and both total and LDL-cholesterol, in patients with a GD of relatively recent onset, suggesting a role of cholesterol in the development of GO (77). Moreover, a correlation was found between CAS and total as well as LDL-cholesterol in untreated GO patients depending on GO duration, indicating a role of cholesterol on GO activity. In the former population of patients with a GD of recent onset, based on the presence or absence of GO, cut-off values were established for total cholesterol at 191 mg/dl and for LDL-cholesterol at 118.4 mg/dL (77). Cholesterol levels above these values were associated with a significantly increased risk of GO. Still in the former population, the percentage of patients with high total cholesterol was significantly greater in patients with GO (77). Overall, the fact that the relation between GO and cholesterol was restricted only to patients with GD of recent onset could be somehow anticipated. In GD patients, there is a close temporal relation between the occurrences of hyperthyroidism and GO, and GO only very rarely appears more than 12 months after the onset of hyperthyroidism (78). This implies that risk factors for GO are more readily identified in patients with a GD of recent onset compared to patients with a long-standing disease, in whom GO occurrence is a very rare event (78). In this context, it is interesting to note that the observations of Stein et al., who, as mentioned above, found a protective role of statins in terms of GO development, were obtained in patients with GD of recent onset (51). In patients with GO, the severity and activity of the eye disease appeared to be minimally affected by serum lipids, even though CAS values were significantly higher in GO patients with high total cholesterol. It is possible that the findings may have been compromised by the fact that some patients had undergone intravenous glucocorticoid treatment for GO before enrollment, with a consequent improvement of GO. Therefore, the analysis was restricted to untreated GO patients, and a significant correlation was found by multivariate analysis with adjustment for GO duration (a variable that correlated with CAS by univariate analysis) between CAS and both total and LDL-cholesterol. In this subgroup of patients and in accordance with the findings obtained in the total cohort of GO patients, CAS was significantly higher in those with high total cholesterol (77). Overall, these data suggest the possibility that not only cholesterol is a possible GO risk factor, but also that it may be associated with more active forms of the eye disease.

In confirmation of these data, in a recent retrospective study, it was reported that patients with GO had higher LDL-cholesterol and total cholesterol compared with patients affected with GD without GO. Furthermore, the percentage of patients who had LDL-cholesterol and total cholesterol levels above the upper limit of the normal range was significantly and remarkably greater in patients with GO (79). These observations suggest that cholesterol is indeed a risk factor for GO.

The mechanisms underlying the potential link between GO and cholesterol may reflect the known altered inflammatory state of hypercholesterolemia, as shown in Figure 1. Thus, the disorders of lipid profile are associated with a mild-to-moderate, systemic, chronic inflammation (36, 37). In hepatocytes, the increased load of free fatty acids is followed by an altered function of mitochondria and endoplasmic reticulum, which ultimately causes the release of reactive oxygen species. Moreover, though indirect mechanisms, free fatty acids trigger the release of pro-inflammatory cytokines, in particular interleukin-6 and tumor-necrosis factor-α, which are both involved in the pathogenetic mechanisms of GO. In support of an altered inflammatory state in hypercholesterolemia as the possible cause of the association between GO and high cholesterol, Busnelli et al. reported that statins are protective in terms of systemic inflammation in swine (80). Obviously, these explanations are speculative and further investigations are required.

## Strengths and Limitations

The observation of a protective role of statins in developing GO in GD patients as well as the correlation of GO and its activity with serum cholesterol are quite novel, and promising for a possible use of statins in GO. On the other hand, the studies available so fare are quite few, no randomized clinical trial are available, because of which the role of statins, and of cholesterol in GO must be considered still preliminary.

## Conclusions

Both basic and clinical findings regarding the effects of statins on GO are encouraging and deserve to be better investigated. Whether statins have an intrinsic role or their effects are related to their capability of lowering cholesterol has to be clarified. Nevertheless, the relation between the presence and the activity of GO and the levels of cholesterol in patients with GD and GO of recent onset may have important clinical implications as they may unravel a new field in GO management, and prompt the necessity for investigating if lowering cholesterol is associated with improved GO outcomes. If the data were confirmed, the use of statins could be considered to improve the efficacy of immunosuppressive therapy, maybe allowing to reduce GC dosage of and consequently limiting their side effects. Clearly, these are just speculations and further, possibly prospective studies are needed.

## Author Contributions

GL conceived and wrote the manuscript. GV, II, IC, FS, and EL performed the literature search and revised manuscript critically. MM conceived, revised, and supervised the manuscript.

## Conflict of Interest

The authors declare that the research was conducted in the absence of any commercial or financial relationships that could be construed as a potential conflict of interest.
